# Dental Consultation in Initially Unknown-Source *Staphylococcus aureus* Bacteremia: A Retrospective Single-Center Cohort Study

**DOI:** 10.3390/antibiotics15060546

**Published:** 2026-05-29

**Authors:** Laura Isabell Werneburg, Ramona Schweyen, Karl-Stefan Delank, Felix Werneburg

**Affiliations:** 1 Department of Prosthodontics and Geriatric Dentistry, Martin Luther University Halle-Wittenberg, 06108 Halle (Saale), Germany; laura.werneburg@uk-halle.de (L.I.W.);; 2 Department of Orthopedic and Trauma Surgery, Martin Luther University Halle-Wittenberg, 06108 Halle (Saale), Germany

**Keywords:** *Staphylococcus aureus* bacteremia, dental consultation, source investigation, unknown-source bacteremia, oral cavity, retrospective cohort study

## Abstract

**Background/Objectives:** Identifying the source of *Staphylococcus aureus* bacteremia (SAB) is central to therapeutic management, but data on dental consultation in initially unknown-source SAB are scarce. We characterized its use, timing, and diagnostic yield, and described the underlying clinical findings and management recommendations through structured re-adjudication. **Methods:** Exploratory retrospective single-center cohort study of adult inpatients with blood-culture-confirmed SAB during 2025, classified at baseline as having an alternative-presumed-source or initially unknown-source SAB. A possible dental focus was defined as a dental condition exhibiting clinical signs of active infection at structured intraoral examination—regardless of whether the active infectious component arose on an acute or, more frequently, on a chronic structural substrate. In the absence of microbiological or molecular confirmation of an odontogenic origin, such findings were interpreted as a possible portal of entry in initially unknown-source patients and as concurrent oral pathology in patients with an alternative-presumed-source. Transesophageal echocardiography (TEE) and infective endocarditis were analyzed as contextual variables; infective endocarditis was extracted as documented by the treating team and was not centrally readjudicated against the modified Duke criteria. **Results:** Of 72 eligible patients, 53 (73.6%) had an alternative-presumed-source and 19 (26.4%) an initially unknown-source SAB. TEE was performed in 54 (75.0%) and infective endocarditis was diagnosed in nine (12.5%) patients, with similar rates in both subgroups. Dental consultation was requested in 17 patients (23.6%), including six of 19 with initially unknown-source SAB (31.6%); a possible dental focus was identified in five of six consulted unknown-source patients (83.3%; 95% CI 43.6–97.0) versus four of 11 alternative source patients (36.4%). This estimate reflects the yield among consultation-selected patients and is not generalizable to the wider unknown-source population. Cardiac and dental evaluation jointly contributed to source clarification in six of 19 unknown-source patients (31.6%). Structured re-adjudication by a blinded dental specialist showed findings dominated by chronic structural dental disease with active inflammatory components rather than classical acute odontogenic infection; active dental treatment was recommended in 11 of 17 patients (64.7%), including all unknown-source patients. **Conclusions:** Dental consultation was performed infrequently in SAB, yet among consultation-selected unknown-source patients it frequently identified clinically suspected oral foci and prompted concrete management, complementing echocardiographic evaluation. Given the exploratory single-center design and the absence of microbiological confirmation of an odontogenic origin, these findings should be interpreted as hypothesis-generating and warrant prospective evaluation with predefined dental criteria and linkage to bacteremia-relevant clinical outcomes.

## 1. Introduction

*Staphylococcus aureus* bacteremia (SAB) remains one of the most consequential bloodstream infections in contemporary clinical practice. Reported in-hospital and 30-day mortality rates of SAB range between 15% and 30%, underscoring its clinical significance as a bloodstream infection syndrome with substantial morbidity and mortality [1,2]. Its relevance derives not only from substantial mortality, but also from its marked propensity for deep-seated and metastatic infection, including infective endocarditis, vertebral osteomyelitis, septic arthritis, and device-associated infection. Accordingly, SAB cannot be approached as an isolated microbiological event; rather, it demands a structured diagnostic strategy aimed at identifying both the portal of entry and clinically relevant secondary foci, alongside timely source control whenever feasible [1,2].

This diagnostic imperative is reflected in current management frameworks, which emphasize repeat blood cultures, echocardiographic evaluation—including systematic assessment against the modified Duke criteria when infective endocarditis is suspected—careful serial clinical assessment, and targeted imaging based on symptoms or risk profile [1,2,3]. In parallel, bedside infectious diseases consultation is associated with more complete evaluation, greater adherence to recommended care processes, and improved recognition of endocarditis and metastatic infection [4,5]. More recently, advanced imaging modalities such as magnetic resonance imaging and 18F-FDG-PET/CT have further expanded the capacity to detect otherwise occult infectious foci in SAB, underscoring how strongly outcome-oriented management depends on systematic source investigation [1,2,6].

In this context, the role of the oral cavity in SAB remains poorly characterized. In infective endocarditis, dental assessment is well established, and contemporary American Heart Association guidance supports comprehensive dental evaluation as part of management when clinically appropriate [3]. Likewise, systematic search strategies for infective endocarditis explicitly include the investigation of potential portals of entry, including the oral cavity [7]. By contrast, in SAB without confirmed endocarditis, dental consultation is not typically framed as a routine element of diagnostic work-up in major clinical reviews [1,2]. This contrast is notable because the broader diagnostic philosophy of SAB is otherwise built around the careful identification of treatable and potentially removable foci, including those that may initially appear clinically unapparent [1,2,3,7].

Part of this uncertainty likely reflects long-standing microbiological assumptions. Although *S. aureus* has not traditionally been considered a major member of the indigenous oral flora, several studies show that it can colonize the oral cavity—including saliva, plaque, and mucosal surfaces—even in otherwise healthy individuals [8,9], and hospital-based screening suggests that the oral cavity may represent a relevant nosocomial reservoir alongside established nasal carriage [10]. Subgingival staphylococci are enriched in diseased periodontal niches and may carry virulence and antimicrobial resistance determinants relevant to invasive infection [11,12,13], and paired oral and nasal isolates show substantial lineage concordance including methicillin-resistant strains, supporting the oral cavity as an under-recognized reservoir of antimicrobial-resistant *S. aureus* rather than a passive extension of nasal carriage [14,15]. Mucosal disruption during routine oral hygiene, dental procedures, and chronic periodontal inflammation can produce transient bacteremia, providing a plausible mechanism by which oral pathology could seed the bloodstream in susceptible patients. Collectively, these findings do not implicate the oral cavity as a common SAB source, but they provide biological plausibility that active dental or periodontal disease may act as an occult portal of entry or as a clinically relevant co-focus.

Despite this biological plausibility and the central role of source investigation in SAB, empirical data on how frequently dental consultation is actually integrated into the diagnostic pathway of SAB—and with what diagnostic yield—are essentially absent from the contemporary literature. In particular, no published study has specifically characterized the use of dental consultation in patients with initially unknown-source SAB, who represent the subgroup in which such evaluation might plausibly contribute the most. Equally unexplored is the clinical content of these consultations: the spectrum of oral pathology encountered, the type and frequency of management recommendations issued, and the extent to which dental assessment leads to concrete in-hospital intervention. We therefore conducted a retrospective single-center cohort study to characterize the use, timing, and diagnostic yield of dental consultation in adult inpatients with SAB, with particular emphasis on those with initially unknown-source SAB, and complemented this with a structured re-adjudication of dental consultation records by an experienced dentist to describe the underlying clinical findings and the management pathways that dental consultation generates in routine SAB care.

## 2. Results

### 2.1. Cohort Characteristics

**Figure 1:**
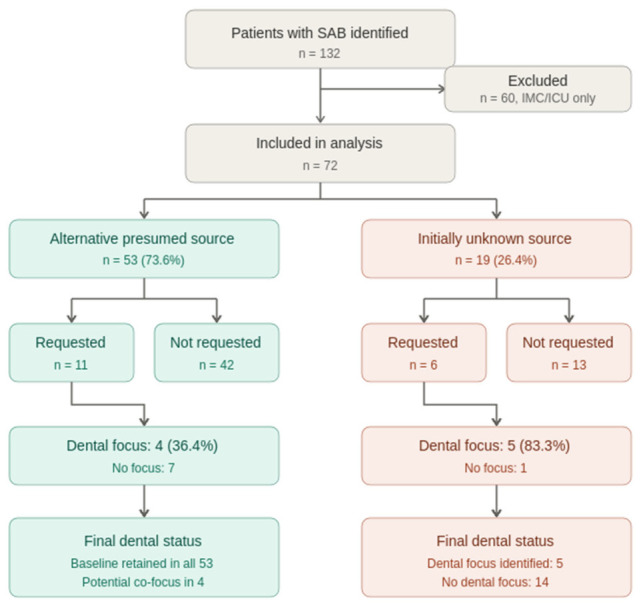
Flow diagram of patients with *Staphylococcus aureus* bacteremia (SAB) according to baseline source status, dental consultation, dental consultation findings, and dental status at discharge or transfer.

A total of 72 adult patients with blood-culture-confirmed *Staphylococcus aureus* bacteremia (SAB) were included in the analysis (Table 1). The median age was 70 years (IQR 62–82), and 41 of 72 patients (56.9%) were male. Most cases were caused by methicillin-susceptible *S. aureus* (MSSA; 70/72, 97.2%), whereas two of 72 cases (2.8%) were methicillin-resistant *S. aureus* (MRSA). Before dental evaluation, 53 of 72 patients (73.6%) had an alternative presumed source of SAB documented, whereas 19 of 72 patients (26.4%) had initially unknown-source SAB. Baseline source categories comprised bone and soft tissue infection in 22 of 72 patients (30.6%), pneumonia in 18 of 72 (25.0%), catheter-associated infection in 10 of 72 (13.9%), urinary tract infection in three of 72 (4.2%), and initially unknown source in 19 of 72 (26.4%). Data completeness was high across key variables. Timing information on TEE was missing for one of the 54 patients in whom it was performed; all other variables relevant to the primary and secondary outcomes were complete. The flow of patients from baseline source classification to dental consultation findings and final source status is summarized in Figure 1.

### 2.2. Echocardiographic Evaluation and Infective Endocarditis

TEE was performed in 54 of 72 patients (75.0%; CI 63.9–83.6). Among patients with an alternative presumed source, TEE was performed in 41 of 53 patients (77.4%; 95% CI 64.5–86.5), whereas among patients with initially unknown-source SAB, TEE was performed in 13 of 19 (68.4%; 95% CI 46.0–84.6). In patients who underwent TEE, the median time from SAB confirmation to TEE request was 2.0 days (IQR 0–2), and the median time from TEE request to completion was 3.0 days (IQR 2–7).

Infective endocarditis was diagnosed in nine of 72 patients overall (12.5%; 95% CI 6.7–22.1). Among patients with an alternative presumed source, infective endocarditis was diagnosed in seven of 53 (13.2%; 95% CI 6.5–24.8), and among patients with initially unknown-source SAB in two of 19 (10.5%; 95% CI 2.9–31.4). Among all TEE-evaluated patients, infective endocarditis was diagnosed in nine of 54 (16.7%; 95% CI 9.0–28.7).

### 2.3. Use of Dental Consultation According to Baseline Source Status

Overall, dental consultation was requested in 17 of 72 patients (23.6%; 95% CI 15.3–34.6). Among patients with initially unknown-source SAB, dental consultation was requested in six of 19 cases (31.6%; 95% CI 15.4–54.0). Among patients with an alternative presumed source documented before dental evaluation, dental consultation was requested in 11 of 53 cases (20.8%; 95% CI 12.0–33.5).

### 2.4. Dental Consultation Findings and Final Source Status in Initially Unknown-Source SAB

Among the six patients with initially unknown-source SAB who underwent dental evaluation, a possible dental focus was identified in five of six cases (83.3%; 95% CI 43.6–97.0). Alongside the dental work-up, infective endocarditis was diagnosed in two of the 19 initially unknown-source patients (10.5%): one of these patients underwent both TEE and dental consultation and had coexisting infective endocarditis and an identified dental focus, whereas the second patient received a TEE-based endocarditis diagnosis without dental consultation.

Taken together, cardiac and dental evaluation contributed to source clarification in six of 19 initially unknown-source patients (31.6%; 95% CI 15.4–54.0): a possible dental focus, interpreted in this subgroup as a possible portal of entry, was identified in five patients (26.3%), infective endocarditis was diagnosed in two patients (10.5%), and one of these patients had both findings concurrently. The remaining 13 of 19 patients (68.4%; 95% CI 46.0–84.6) remained without an identified source at discharge or transfer. No patient with initially unknown-source SAB was subsequently assigned to a different non-dental, non-cardiac source in the discharge or transfer documentation.

### 2.5. Dental Consultation Findings in Patients with an Alternative Presumed Source at Baseline

Among the 11 patients with an alternative presumed source already documented before dental evaluation who underwent dental consultation, a possible dental focus was identified in four of 11 cases (36.4%; 95% CI 15.2–64.6). In all four cases, the baseline source attribution was retained in the discharge or transfer documentation, and the dental finding was recorded as concurrent oral pathology—i.e., a potential co-focus—rather than a basis for primary source reclassification. None of the seven patients with diagnosed infective endocarditis in this subgroup underwent dental consultation; the four identified dental foci therefore occurred exclusively in non-endocarditis patients, and the alternative source yield of dental consultation is not attributable to endocarditis-driven portal-of-entry evaluation. Across all 17 patients who underwent dental evaluation, a possible dental focus was identified in nine of 17 cases (52.9%; 95% CI 31.0–73.8). These outcomes are summarized in Table 2.

### 2.6. Timing of Dental Evaluation

Across all 17 patients who underwent dental evaluation, the median time from microbiological report release confirming SAB to dental consultation request was 3.0 days (IQR 2–6; range 0–22), and the median time from consultation request to completed dental evaluation was 3.0 days (IQR 1–5; range 1–11). In the alternative presumed source subgroup (*n* = 11), the median time from SAB confirmation to consultation request was 3.0 days (IQR 1.5–7), and the median time from request to completion was 3.0 days (IQR 1.5–5). In patients with initially unknown-source SAB who underwent dental evaluation (*n* = 6), the median time from SAB confirmation to consultation request was 3.5 days (IQR 3–4), and the median time from request to completion was 3.5 days (IQR 1.5–9).

### 2.7. Clinical Spectrum and Management Recommendations Derived from Dental Consultation

Structured re-adjudication was performed for all 17 completed dental consultations. The most frequently documented clinical findings were poor oral hygiene in nine of 17 patients (52.9%; 95% CI 31.0–73.8), suspected periodontitis in seven of 17 (41.2%; 95% CI 21.6–64.0), and deeply penetrating caries in seven of 17 (41.2%; 95% CI 21.6–64.0). Less frequently documented findings included residual roots in four of 17 (23.5%; 95% CI 9.6–47.3), non-salvageable teeth in three of 17 (17.6%; 95% CI 6.2–41.0), apical lesions in two of 17 (11.8%; 95% CI 3.3–34.3), and mucosal ulceration in one of 17 (5.9%). Acute inflammatory signs such as fistulae, abscesses, pericoronitis, or peri-implant inflammation were not documented in any of the 17 consultations. Dental imaging was performed during the consultation in six of 17 patients (35.3%).

Active dental treatment—encompassing conservative, surgical, or periodontal intervention—was recommended in 11 of 17 patients (64.7%; 95% CI 41.3–82.7). Considered separately, conservative treatment was recommended in nine of 17 (52.9%), surgical treatment in five of 17 (29.4%), and periodontal treatment in six of 17 (35.3%). Further diagnostic work-up was recommended in six of 17 (35.3%), additional imaging in eight of 17 (47.1%), and outpatient dental follow-up in seven of 17 (41.2%). Immediate in-hospital dental treatment was performed during the current admission in four of 17 patients (23.5%; 95% CI 9.6–47.3).

**Figure 2:**
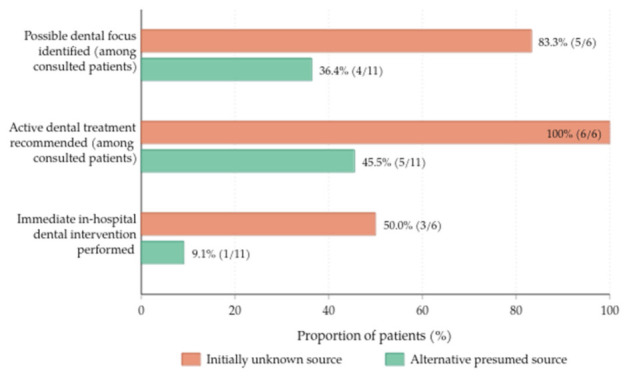
Stratified diagnostic and management outcomes of dental consultation in *Staphylococcus aureus* bacteremia.

When stratified by baseline source status, active dental treatment was recommended in all six patients with initially unknown-source SAB (100%; 95% CI 61.0–100.0) compared with five of 11 patients with an alternative presumed source (45.5%; 95% CI 21.3–72.0). Immediate in-hospital dental intervention was performed in three of six initially unknown-source patients (50.0%) and in one of 11 alternative source patients (9.1%). The underlying clinical findings were consistently in the domain of chronic structural dental disease with active inflammatory components—predominantly poor oral hygiene, suspected periodontitis, and deeply penetrating caries—rather than classical acute odontogenic infection, in both subgroups. These findings are summarized in Table 3 and visualized in Figure 2.

## 3. Discussion

In this retrospective single-center cohort study, dental consultation was requested in fewer than one-quarter of all patients with *Staphylococcus aureus* bacteremia and in less than one-third of those with initially unknown-source SAB. Yet among consulted initially unknown-source patients, a possible dental focus was identified in the majority, and structured re-adjudication of all 17 consultation records revealed that nearly two-thirds of consulted patients—and every initially unknown-source patient—received a recommendation for active dental intervention. Considered together, these findings suggest that dental consultation is not routinely integrated into the diagnostic work-up of SAB, but that when performed selectively in patients without an otherwise identifiable source it can detect clinically suspected oral foci and translate into concrete management pathways. The relatively high proportion of focus identification in this group reflects the yield in consultation-selected patients and should not be misread as a population-level prevalence of dental foci in unknown-source SAB.

This observation is clinically relevant because SAB management is fundamentally structured around source investigation. Contemporary reviews and care frameworks emphasize repeated blood cultures, echocardiographic assessment, serial clinical reassessment, and targeted imaging to identify both the portal of entry and metastatic foci of infection [1,2,16]. Infectious diseases consultation has likewise been associated with more complete diagnostic evaluation and improved adherence to key management processes in SAB [4,5], and advanced imaging modalities such as magnetic resonance imaging further expand the capacity to detect occult foci that would otherwise remain unrecognized [6]. Against this backdrop, our findings draw attention to a notable inconsistency: while the search for hidden foci is central to modern SAB care, dental evaluation is not generally conceptualized as a routine component of this diagnostic pathway, despite its established role in infective endocarditis and broader portal-of-entry assessment [3,7].

A central interpretive lens for our findings is the concurrent echocardiographic work-up. TEE was performed in 75.0% of patients overall and in 68.4% of those with initially unknown-source SAB, reflecting active adherence to established SAB care. Infective endocarditis was diagnosed in 10.5% of the initially unknown-source subgroup (two of 19 patients), a rate similar to that observed in the alternative presumed source subgroup (13.2%). Cardiac evaluation therefore contributed to possible source clarification in a minority of initially unknown-source patients. When cardiac and dental evaluation were combined, a possible source was suggested in approximately one-third of initially unknown-source patients, while two-thirds remained without an identified source at discharge or transfer. The two patient groups suggested by each approach were largely non-overlapping; only a single patient presented with both a possible dental portal of entry and infective endocarditis, in whom the dental lesion is clinically plausible as a portal of entry. This pattern is consistent with cardiac and dental evaluation acting as complementary diagnostic modalities in unknown-source SAB, and supports the view that the oral cavity should be considered more explicitly when standard work-up fails to identify a plausible source, without in any way displacing echocardiographic assessment.

The interpretation of dental findings in patients who already had an alternative source documented before dental evaluation merits particular attention. A possible dental focus was identified in four of the 11 consulted patients in this subgroup, corresponding to a yield of 36.4%. In all four cases, the baseline source attribution was retained in the discharge documentation, and the dental finding was recorded as concurrent oral pathology—i.e., a potential co-focus—rather than as a primary source. Notably, none of the seven patients with diagnosed infective endocarditis in this subgroup underwent dental consultation; the four identified dental foci therefore occurred exclusively in non-endocarditis patients, and the alternative source yield cannot be attributed to guideline-driven portal-of-entry evaluation in endocarditis care. These findings should nonetheless be interpreted with caution, as the identification of an active oral pathology in a patient with an already documented alternative source of bacteremia does not, in itself, establish a causal contribution of the dental finding to the bacteremic episode; the four cases may equally represent either a true co-focus or active oral pathology unrelated to the index bacteremia. While they support the diagnostic relevance of dental consultation in selected patients, they do not justify routine reclassification of source attribution on the basis of abnormal dental findings alone in the absence of stronger corroborating evidence.

The structured re-adjudication of dental consultation records adds a mechanistic dimension that is not visible in the binary focus classification alone. The dominant findings were in the domain of chronic structural dental disease with active inflammatory components—poor oral hygiene in 52.9% of consulted patients, suspected periodontitis in 41.2%, and deeply penetrating caries in 41.2%, often in association with infected residual roots or non-salvageable teeth—whereas classical acute odontogenic pathology such as acute apical inflammation, abscesses, fistulae, or pericoronitis was entirely absent. This pattern is consistent with contemporary data implicating subgingival *S. aureus* as an organism enriched in diseased periodontal niches rather than in acutely infected sites [11,12,13], and it aligns with the biological rationale that, if the oral cavity contributes to SAB pathogenesis, the more plausible mechanism is persistent mucosal and periodontal inflammation—creating a sustained reservoir and repeated low-grade mucosal breach—rather than acute suppurative odontogenic infection. From a clinical standpoint, these observations generate the hypothesis that the target population for dental evaluation in SAB may warrant reconsideration: rather than concentrating exclusively on patients with overt acute odontogenic infection, dental evaluation may be most informative in patients with chronic structural dental disease exhibiting clinical signs of active inflammation, in whom recurrent low-grade bacteremic seeding is a biologically plausible mechanism that warrants prospective characterization.

This pattern of findings warrants careful but not dismissive interpretation. Chronic structural dental disease—particularly chronic periodontal disease, residual roots, deeply penetrating caries, and poor oral hygiene—is highly prevalent among elderly hospitalized patients, and dental examiners specifically searching for oral pathology in this population can reasonably be expected to identify such conditions at high frequency irrespective of bacteremic status. Importantly, however, the present analysis did not classify chronic dental disease as a possible focus on the basis of chronicity alone; the focus classification required clinical signs of active infection—most frequently in the form of suppuration in advanced periodontitis, infected residual roots, or periapical pathology consistent with active infection—arising on a chronic structural substrate. The biological plausibility of chronic active oral inflammation as a source of recurrent low-grade bacteremia—through repeated mucosal breach during routine oral hygiene, mastication, and dental procedures—is well established, and the pattern of findings in our cohort is consistent with this mechanism. Chronic oral inflammatory disease with active infectious components should therefore be regarded as a plausible source candidate in SAB, particularly when no alternative source has been identified. What remains constrained, in the absence of microbiological or molecular linkage between oral and blood culture isolates, is the individual causal attribution at the patient level—that is, the question of whether the oral lesion identified in a given patient is indeed the source of that patient’s bacteremia. This distinction—between mechanistic plausibility at the population level, which our findings support, and causal attribution at the individual patient level, which our findings cannot establish—underscores the value of prospective evaluation incorporating microbiological linkage between oral and blood culture isolates.

Beyond diagnostic yield, the actionability of dental consultation emerges as a clinically important dimension of our findings. Across all consulted patients, active dental treatment—whether conservative, surgical, or periodontal—was recommended in 64.7% of patients overall and in every initially unknown-source patient (six out of six). Immediate in-hospital dental intervention was performed in 23.5% of consulted patients, including 50.0% of initially unknown-source patients. These observations suggest that dental consultation in SAB may extend beyond a purely diagnostic exercise in portal-of-entry assessment and may, in selected patients, lead to management recommendations with potential clinical implications. Both the rate of active treatment recommendations and the rate of immediate in-hospital intervention were higher in the initially unknown-source subgroup, paralleling the higher focus yield in this group. This pattern raises the possibility that dental consultation in patients without an alternative plausible source may contribute to both diagnostic clarification and the identification of actionable management pathways. Whether implementing these recommendations translates into improved bacteremia outcomes, reduced relapse rates, or altered antimicrobial management cannot be answered from the present data and represents a direct priority for prospective investigation.

The timing of consultation is similarly notable. Across all patients who underwent dental evaluation, dental consultation was requested only after a median delay of 3 days from microbiological confirmation, with a further 3-day median interval until in-person examination, corresponding to approximately 6 days in total until a dental focus could be detected or excluded. In patients with initially unknown-source SAB, this interval extended to approximately 7 days. These timelines compare unfavorably with those of TEE in the same cohort, for which the median interval from SAB confirmation to TEE request was 2 days. In a syndrome in which early source identification and timely source control are key management principles, this pattern suggests that dental evaluation was generally incorporated late, if at all, into the diagnostic pathway, and later than other established diagnostic modalities. Although our study cannot determine whether earlier dental consultation would improve source identification or clinical outcomes, the observed delay supports the impression that dental evaluation currently functions more as an optional downstream measure than as an integrated early component of SAB work-up.

A further interpretive caveat concerns the 13 initially unknown-source patients who did not undergo dental evaluation. Twelve of these patients remained without an identified source at discharge or transfer; in the remaining patient, infective endocarditis was diagnosed through echocardiography in the absence of dental consultation. Because the decision to request dental evaluation was not protocolized but left to the discretion of the treating team, this introduces a substantial risk of confounding by indication: patients referred for dental evaluation may have been selected on the basis of unmeasured factors such as clinical suspicion of oral pathology, cooperativeness, mobility, or anticipated length of stay. The 83.3% focus yield observed among consulted initially unknown-source patients should therefore be interpreted as the yield in consultation-selected patients rather than as a population-level prevalence of dental foci in unknown-source SAB; the true proportion of patients with an oral focus in the wider unknown-source population is very likely to be lower, potentially considerably so. The observed association between dental consultation and source identification in the initially unknown-source subgroup therefore cannot be interpreted as a causal effect of dental consultation itself. Nevertheless, the fact that no alternative source was identified in the majority of patients who did not undergo dental evaluation underscores that, under current practice, the absence of a dental work-up in initially unknown-source SAB is associated with persistent diagnostic uncertainty.

Several limitations must be acknowledged. First, this was a retrospective single-center study with a modest sample size, and the subgroup of patients with initially unknown-source SAB who underwent dental evaluation was small (*n* = 6); consequently, the corresponding point estimates are accompanied by wide confidence intervals and should be considered exploratory and hypothesis-generating. Second, patients managed exclusively in intermediate or intensive care settings (*n* = 60; 45.5% of the initially identified source cohort) were excluded because of structural differences in clinical documentation, the limited feasibility of structured dental examination in sedated or intubated patients, and a consultation pathway in which dental evaluations are performed predominantly as outpatient referrals to the dental clinic rather than as bedside consultations in critical care wards; the role of dental evaluation in this distinct critically ill population therefore remains an open question for separate investigation. Third, dental consultation was not protocolized but left to the discretion of the treating team, introducing a substantial risk of selection bias and confounding by indication, as discussed above. Fourth, the classification of a “possible dental focus” was based on clinical dental documentation rather than on microbiological confirmation, molecular matching of blood and oral isolates, or predefined source adjudication criteria specific to odontogenic infection; furthermore, consultations were performed by the respective dental specialist on duty without formal inter-examiner calibration, no specialty-specific written examination protocol was in place during the study period, and inter-rater reliability was therefore not assessed. Accordingly, the term “possible dental focus” denotes a clinically suspected, not a microbiologically proven, source. Fifth, the diagnosis of infective endocarditis was made by the treating clinical department and could not be centrally readjudicated against current consensus criteria—i.e., the modified Duke or the more recent 2023 Duke–ISCVID criteria [17]—in this retrospective dataset; this methodological caveat is symmetric to the dental focus classification—neither modality was centrally validated against a predefined reference standard—and limits the precision of cardiac-versus-dental comparisons. Finally, we did not systematically collect data on oral symptoms, baseline dental status, persistent bacteremia, relapse, in-hospital and 30-day mortality, or changes to antimicrobial management triggered by dental findings; outcome-based inference, including any assessment of whether dental evaluation altered the clinical course of SAB, is therefore not possible from the present dataset and is identified as a direct priority for prospective follow-up work.

Notwithstanding these limitations, the study has several methodological strengths. We examined a clearly defined cohort of adult patients with microbiologically confirmed SAB, used independent retrospective source adjudication, reported all proportions with 95% confidence intervals, and separated baseline source status from dental consultation findings and final source status at discharge or transfer. This temporal distinction is methodologically important because it avoids conflating initially unknown-source SAB with cases that became only partially clarified after downstream diagnostic evaluation. By analyzing dental consultation alongside the concurrent echocardiographic work-up and the observed endocarditis rate, we provide a broader diagnostic picture that more realistically represents real-world inpatient SAB management. A further strength is the structured re-adjudication of dental consultation findings by an experienced dentist blinded to baseline source group allocation, which allowed characterization of the clinical spectrum and management recommendations beyond the binary focus classification used in routine documentation. Finally, the study reflects real-world inpatient practice and therefore provides pragmatic information not only on diagnostic yield but also on how infrequently and how late dental consultation is currently incorporated into SAB work-up.

The generalizability of our findings is constrained by several design features. The study was conducted at a single German tertiary care center using diagnostic and coding frameworks specific to the German DRG system, and the findings reflect inpatient SAB management in general wards rather than in intensive care settings. The extent to which the observed patterns of dental consultation use, timing, and diagnostic yield translate to other healthcare systems, consultation structures, or clinical settings therefore requires confirmation in multicenter studies with diverse institutional contexts.

From a clinical perspective, our data do not support routine dental consultation for all patients with SAB. They do, however, support a lower threshold for dental evaluation in selected patients with initially unknown-source SAB, particularly when standard diagnostic work-up, including echocardiography, has failed to identify a plausible source and when oral pathology is clinically suspected. These observations also have potential implications for antibiotic management strategies in SAB. Recent work has explored eligibility criteria for early intravenous-to-oral antibiotic switch in selected SAB patients [18,19], a strategy that critically depends on confident exclusion or treatment of all plausible foci of infection. Inadequately characterized dental foci could compromise such stewardship strategies, providing additional rationale for systematic source investigation that includes dental evaluation in patients without an alternative source. Whether a more systematic and earlier dental assessment strategy can improve source identification, source control, or clinically meaningful outcomes—including duration of bacteremia, frequency of persistent bacteremia, relapse, in-hospital and 30-day mortality, and dental-finding-driven changes to antimicrobial management—remains unknown and should be addressed in prospective studies using predefined dental criteria, standardized examination protocols, central re-adjudication of infective endocarditis against current consensus criteria (i.e., the modified Duke or 2023 Duke–ISCVID criteria [17]), and microbiological linkage between oral and blood culture isolates where feasible. Future studies might also evaluate digital tools and decision-support systems—including telehealth-based dental triage [20] and dental antimicrobial stewardship applications [21]—to support timely and standardized identification of dental foci in inpatients with SAB, particularly in care environments with limited bedside dental access.

## 4. Materials and Methods

### 4.1. Study Design and Settings

We conducted a retrospective single-center cohort study at a tertiary care hospital including adult inpatients treated for *Staphylococcus aureus* bacteremia (SAB) between 1 January 2025 and 31 December 2025. The study was reported in accordance with the Strengthening the Reporting of Observational Studies in Epidemiology (STROBE) guidelines for cohort studies.

### 4.2. Study Population and Case Ascertainment

The study population comprised adult patients with one distinct episode of blood-culture-confirmed SAB during the study period. The unit of analysis was the individual patient. Initial case identification was performed through the hospital’s case management department using a combined query of the German Modification of the International Classification of Diseases, 10th Revision (ICD-10-GM), and the German Operation and Procedure Classification (OPS). All potentially eligible cases were subsequently reviewed in the institutional electronic medical record system (ORBIS; Dedalus HealthCare, Bonn, Germany), and microbiological confirmation of SAB was verified manually on the basis of the original blood culture report. Only patients with confirmed *S. aureus* bacteremia during the respective inpatient stay were included. Patients treated exclusively in intermediate care (IMC) or intensive care unit (ICU) wards were excluded after manual chart review for three structural reasons: First, IMC and ICU wards at our institution use a separate clinical information system, which does not allow the same level of structured retrospective ascertainment of dental consultation records as the electronic medical record system used in general wards. Second, formal dental consultation in critically ill, frequently sedated, or intubated patients is uncommon in routine practice, as a structured intraoral examination is rarely feasible under these conditions. Third, dental consultations at our institution are predominantly performed as outpatient referrals to the Department of Dental, Oral and Maxillofacial Medicine; bedside consultations in IMC or ICU wards are not part of the routine consultation pathway. Patients who were transferred to a general ward at any point during their inpatient stay and met all inclusion criteria were retained in the cohort. A total of 132 adult inpatients with blood-culture-confirmed SAB were initially identified during the study period. Of these, 60 (45.5%) were excluded because they had been treated exclusively in intermediate care or intensive care wards, leaving 72 patients eligible for inclusion in the present analysis. This exclusion represents a substantial proportion of the initial source cohort and introduces a structural selection that warrants consideration when interpreting the findings: the present analysis characterizes dental consultation practice in general wards and does not generalize to critically ill SAB patients managed in intensive care settings.

### 4.3. Source Adjudication

The presumed source of SAB before dental evaluation was adjudicated retrospectively and independently by two investigators using all electronically available clinical documentation in ORBIS, including physician notes, discharge summaries, microbiological data, and diagnostic work-up results. Source assignment was based on the documented clinical assessment and available diagnostic findings, including, where applicable, imaging and other clinically obtained investigations. Cases with discrepant or unclear classification were reviewed jointly and resolved by consensus.

For the purpose of this analysis, patients were classified at baseline as having either an alternative presumed source of SAB or initially unknown-source SAB. Initially unknown-source SAB was defined as the absence of a plausible documented source before dental evaluation. Final source status was assessed separately on the basis of the discharge or transfer documentation at the end of the hospital stay.

### 4.4. Echocardiographic Evaluation and Diagnosis of Infective Endocarditis

Transesophageal echocardiography (TEE) was performed at the discretion of the treating clinical department as part of routine SAB work-up and was not protocolized for the purpose of this study. The timing of TEE request and completion was extracted retrospectively from the electronic medical record. The diagnosis of infective endocarditis was made by the treating clinical department and recorded as documented in the electronic medical record. The application of formal diagnostic frameworks for infective endocarditis—i.e., the modified Duke criteria [3] or, more recently, the 2023 Duke–ISCVID criteria [17]—was at the discretion of the managing team and could not be reliably ascertained retrospectively; accordingly, we report infective endocarditis as clinically documented rather than as centrally readjudicated against a predefined reference standard. This methodological caveat is symmetric to our handling of the dental focus classification (Section 2.5) and should be considered when the two evaluation pathways are compared in the present analysis.

### 4.5. Dental Consultation and Definition of a Possible Dental Focus

Dental consultation was defined as an electronically documented consultation request from the treating team, issued via the institutional electronic medical record system (ORBIS), to the university Department of Dental, Oral and Maxillofacial Medicine. A completed dental consultation required a documented in-person clinical dental examination. Examinations were performed by the dental specialist on duty for inpatient consultations at the time of evaluation, consistent with the institutional consultation workflow; no specialty-specific written consultation protocol or structured intraoral examination template was in place during the study period. Dental imaging during the consultation (e.g., panoramic radiography) was not mandatory and was obtained at the discretion of the examining dentist on the basis of the clinical situation.

A possible dental focus was defined as a dental condition documented during in-person clinical dental examination that the examining dentist considered to represent a plausible infectious source of bacteremia. To distinguish such findings from chronic dental disease without active infectious activity and from incidental oral pathology, three operational categories were applied. First, dental conditions exhibiting clinical signs of active infection—encompassing acute odontogenic abscess, purulent dental or periodontal infection, apical periodontitis or periapical pathology consistent with active infection, advanced periodontitis with clinical signs of active inflammation or suppuration, infected residual roots, pericoronitis, peri-implant infection with signs of active infection, or deeply penetrating caries with pulpal or periapical involvement—were classified as a possible dental focus, regardless of whether the active infectious component arose on an acute or, more frequently, on a chronic structural substrate. Second, chronic dental disease without clinical signs of active infection—including fractured but painless and clinically vital teeth, well-restored teeth without periapical pathology, or chronic periodontal pockets without acute inflammatory signs or suppuration—was not classified as a possible dental focus, even when prevalent. Third, isolated incidental oral findings of unclear infectious relevance were likewise not classified as a possible dental focus. The defining criterion for classification was therefore the presence of clinical signs of active infection, not the chronicity of the underlying dental disease. Because consultations were performed by the respective dental specialist on duty at the time of evaluation, the clinical judgment of a possible dental focus reflects the assessment of multiple examining dentists, and no formal inter-examiner calibration was conducted; inter-rater reliability was therefore not assessed. Because microbiological or molecular confirmation linking oral isolates to blood culture isolates was not available in this dataset, a positive focus classification represents a clinically suspected, not a microbiologically proven, source. To reflect this conceptual hierarchy in the interpretation of the results, dental findings were further qualified as a possible portal of entry when identified in patients with initially unknown-source SAB, and as concurrent oral pathology—i.e., a potential co-focus—when identified in patients with an alternative presumed source documented before dental evaluation. The term probable source of bacteremia, which would require microbiological or molecular corroboration, is not applied to any patient in the present analysis.

### 4.6. Structured Re-Adjudication of Dental Consultation Findings

To characterize the clinical spectrum of dental findings and resulting management recommendations beyond the binary focus classification, we performed a structured re-adjudication of all dental consultation records. Re-adjudication was conducted by an experienced dentist (L.I.W.), blinded to baseline source group allocation, applying a predefined categorical extraction framework. This framework covered (i) clinical findings (apical lesion, acute apical inflammation, fistula, abscess, pericoronitis, deeply penetrating caries, non-salvageable teeth, residual roots, suspected periodontitis, peri-implant inflammation, mucosal ulceration, denture-associated lesions, poor oral hygiene), (ii) dental imaging performed during the consultation, (iii) recommended management (further diagnostic work-up, additional imaging, conservative treatment, surgical treatment, periodontal treatment, outpatient dental follow-up), and (iv) immediate in-hospital dental intervention performed during the current admission. The re-adjudication provides a more granular, standardized description of dental pathology encountered during the consultation and does not replace the original binary focus classification used in the primary analysis; it was completed for all 17 dental consultations.

### 4.7. Data Collection

Clinical and diagnostic data were extracted retrospectively from the electronic medical record. In addition to demographic and microbiological information, variables collected for the present analysis included baseline source classification before dental evaluation, whether dental consultation was requested and completed, the timing of consultation request and completion, the documented result of dental assessment, whether TEE was performed and the corresponding timing, the diagnosis of infective endocarditis as documented by the treating department, and final source status at hospital discharge or transfer.

### 4.8. Outcomes

The primary outcome was the proportion of patients with initially unknown-source SAB in whom a dental consultation was requested. Secondary outcomes were the time from microbiological report release confirming SAB to dental consultation request, the time from dental consultation request to completed dental evaluation, and the proportion of evaluated patients in whom the dental assessment identified a possible dental focus.

Exploratory analyses described the relationship between baseline source status, use of dental consultation, dental consultation findings, and final source status at discharge or transfer. In particular, patients with initially unknown-source SAB were differentiated into those in whom dental consultation identified a possible dental focus and those in whom no possible dental focus was identified and the source remained unknown until discharge or transfer. As contextual variables, we additionally analyzed the use and timing of TEE and the frequency of infective endocarditis, stratified by baseline source status. In addition, clinical findings and management recommendations derived from the structured re-adjudication of dental consultations were summarized descriptively—overall and stratified by baseline source status.

### 4.9. Study Size

The study included all consecutive adult inpatients with blood-culture-confirmed SAB who met the eligibility criteria during the 2025 calendar year. As an exploratory, hypothesis-generating, single-center pilot analysis with a fixed observation window, no a priori sample size calculation was performed; the analysis was designed to estimate proportions with accompanying 95% confidence intervals rather than to test prespecified hypotheses, and the resulting subgroup denominators—in particular the *n* = 6 patients with initially unknown-source SAB who underwent dental consultation—should be interpreted accordingly.

### 4.10. Statistical Analysis

This study was designed as an exploratory retrospective cohort analysis. Categorical variables are reported as counts and percentages with 95% confidence intervals (CI) calculated using the Wilson score method. Continuous variables are reported as median with interquartile range (IQR) and range where informative. The analyses were primarily descriptive given the sample size and exploratory study design; no inferential hypothesis testing was prespecified, and no adjustment for multiplicity was performed. Missing data were reported descriptively for each affected variable; no imputation was performed. No sensitivity analyses were prespecified or conducted given the exploratory study design. Descriptive analyses were conducted using IBM SPSS Statistics (IBM Corp., Armonk, NY, USA), version 31. Wilson 95% confidence intervals for proportions were computed using Python (version 3.12.3; Python Software Foundation, Beaverton, OR, USA).

### 4.11. Ethics

The study was reviewed by the local ethics committee of the Martin Luther University Halle-Wittenberg, which issued a statement of no objection for this retrospective analysis. The study was conducted in accordance with the Declaration of Helsinki.

## 5. Conclusions

Dental consultation was infrequently and belatedly integrated into the diagnostic work-up of *Staphylococcus aureus* bacteremia in our cohort. When performed in patients with initially unknown-source SAB, however, dental evaluation identified a clinically suspected oral focus—interpreted as a possible portal of entry—in the majority of consulted patients and was frequently associated with recommendations for active dental intervention, while the underlying findings were predominantly in the domain of chronic structural dental disease with active inflammatory components rather than classical acute odontogenic infection. Cardiac and dental evaluation appeared to act as complementary diagnostic modalities, jointly contributing to possible source clarification in a subset of initially unknown-source patients. The biological plausibility of chronic active oral inflammation as a source of recurrent low-grade bacteremia is well established, and the pattern of findings in our cohort is consistent with this mechanism. What remains constrained—in light of the small consultation-selected subgroup, the absence of microbiological linkage between oral and blood culture isolates, and the lack of central re-adjudication of infective endocarditis against current consensus diagnostic criteria—is the individual causal attribution at the patient level rather than the mechanistic plausibility itself. These observations should therefore be interpreted as hypothesis-generating: they support chronic oral inflammatory disease with active infectious components as a plausible source candidate in unknown-source SAB and motivate prospective, protocolized evaluation with predefined dental criteria, microbiological linkage where feasible, and prespecified bacteremia-relevant clinical outcomes.

## Figures and Tables

**Table 1:** Baseline characteristics and source-related diagnostic context of the study cohort—overall and stratified by initial source status of *Staphylococcus aureus* bacteremia before dental evaluation.

Characteristic	Overall (*n* = 72)	Alternative Presumed Source (*n* = 53)	Initially Unknown Source (*n* = 19)
Demographics			
Age, years, median (IQR)	70 (62–82)	70 (63–82)	68 (62–82)
Male sex, *n* (%)	41 (56.9)	30 (56.6)	11 (57.9)
**Microbiology**			
MSSA, *n* (%)	70 (97.2)	51 (96.2)	19 (100.0)
MRSA, *n* (%)	2 (2.8)	2 (3.8)	0 (0.0)
**Documented source category at baseline, *n* (%)**			
Bone and soft tissue infection	22 (30.6)	22 (41.5)	—
Pneumonia	18 (25.0)	18 (34.0)	—
Catheter-associated infection	10 (13.9)	10 (18.9)	—
Urinary tract infection	3 (4.2)	3 (5.7)	—
Initially unknown source	19 (26.4)	—	19 (100.0)
**Echocardiographic evaluation**			
Transesophageal echocardiography performed, *n* (%)	54 (75.0)	41 (77.4)	13 (68.4)
Time from SAB confirmation to TEE request, days, median (IQR) *	2.0 (0–2)	1.0 (0–2)	2.0 (1–4)
Time from TEE request to completion, days, median (IQR) *	3.0 (2–7)	3.0 (1–7)	3.0 (3–4)
**Infective endocarditis**			
Infective endocarditis diagnosed, *n* (%)	9 (12.5)	7 (13.2)	2 (10.5)
Among TEE-evaluated patients, *n*/N (%)	9/54 (16.7)	7/41 (17.1)	2/13 (15.4)

* Timing data available for 53 of 54 patients who underwent TEE (40 in the alternative presumed source subgroup, 13 in the initially unknown-source subgroup). Abbreviations: IQR, interquartile range; MRSA, methicillin-resistant *Staphylococcus aureus*; MSSA, methicillin-susceptible *Staphylococcus aureus*; SAB, *Staphylococcus aureus* bacteremia; TEE, transesophageal echocardiography.

**Table 2:** Dental consultation utilization, timing, and diagnostic yield—overall and stratified by initial source status before dental evaluation.

Characteristic	Overall (*n* = 72)	Alternative Presumed Source (*n* = 53)	Initially Unknown Source (*n* = 19)
Utilization			
Dental consultation requested, *n* (%) [95% CI] *	17 (23.6) [15.3–34.6]	11 (20.8) [12.0–33.5]	6 (31.6) [15.4–54.0]
**Timing of dental consultation †**			
Time from SAB confirmation to consultation request, days, median (IQR)	3.0 (2–6)	3.0 (1.5–7)	3.5 (3–4)
Time from consultation request to completion, days, median (IQR)	3.0 (1–5)	3.0 (1.5–5)	3.5 (1.5–9)
**Diagnostic yield**			
Possible dental focus identified, *n*/N (%) [95% CI] ‡	9/17 (52.9) [31.0–73.8]	4/11 (36.4) [15.2–64.6]	5/6 (83.3) [43.6–97.0]
**Final source status at discharge or transfer, *n* (%) [95% CI]**			
Dental focus identified as possible source	9 (12.5) [6.7–22.1]	4 (7.5) [3.0–17.9] §	5 (26.3) [11.8–48.8]
No dental focus identified at discharge or transfer	63 (87.5)	49 (92.5) §	14 (73.7)
Patients without dental consultation, *n*	55	42	13
Patients with dental consultation but no dental focus identified, *n*	8	7	1

* 95% confidence intervals calculated using the Wilson score method. † Timing data reported for all patients with a completed dental consultation. ‡ Denominator refers to patients who underwent dental consultation. § In the alternative presumed source subgroup, possible dental foci were interpreted as potential co-foci rather than primary source reclassification; the baseline source attribution was retained in all cases. Refers to dental focus identification only; concurrent diagnosis of infective endocarditis, where applicable, is reported separately in Table 1. In the initially unknown-source subgroup, infective endocarditis was additionally diagnosed in 2 patients, one of whom also had an identified dental focus. Abbreviations: CI, confidence interval; IQR, interquartile range; SAB, *Staphylococcus aureus* bacteremia.

**Table 3:** Clinical findings and management recommendations derived from structured re-adjudication of dental consultations—overall and stratified by baseline source status. Re-adjudication was conducted by an experienced dentist (L.I.W.), blinded to baseline source group allocation, and covered all 17 completed dental consultations.

Characteristic	All Consultations (*n* = 17)	Alternative Presumed Source (*n* = 11)	Initially Unknown Source (*n* = 6)
Clinical findings, *n* (%)			
Poor oral hygiene	9 (52.9)	6 (54.5)	3 (50.0)
Suspected periodontitis	7 (41.2)	4 (36.4)	3 (50.0)
Deeply penetrating caries	7 (41.2)	3 (27.3)	4 (66.7)
Residual root	4 (23.5)	1 (9.1)	3 (50.0)
Non-salvageable tooth	3 (17.6)	0 (0.0)	3 (50.0)
Apical lesion	2 (11.8)	1 (9.1)	1 (16.7)
Mucosal ulceration	1 (5.9)	0 (0.0)	1 (16.7)
Acute inflammatory pathology *	0 (0.0)	0 (0.0)	0 (0.0)
**Dental imaging, *n* (%)**			
Imaging performed during consultation	6 (35.3)	3 (27.3)	3 (50.0)
**Management recommendations, *n* (%)**			
Further diagnostic work-up recommended	6 (35.3)	4 (36.4)	2 (33.3)
Additional imaging recommended	8 (47.1)	4 (36.4)	4 (66.7)
Conservative treatment recommended	9 (52.9)	3 (27.3)	6 (100.0)
Surgical treatment recommended	5 (29.4)	1 (9.1)	4 (66.7)
Periodontal treatment recommended	6 (35.3)	4 (36.4)	2 (33.3)
Outpatient dental follow-up recommended	7 (41.2)	4 (36.4)	3 (50.0)
Any active dental treatment recommended †,‡	11 (64.7) [41.3–82.7]	5 (45.5) [21.3–72.0]	6 (100.0) [61.0–100.0]
**In-hospital intervention, *n* (%)**			
Immediate dental treatment performed during admission ‡	4 (23.5) [9.6–47.3]	1 (9.1) [1.6–37.7]	3 (50.0) [18.8–81.2]

* Acute inflammatory pathology comprises acute apical inflammation, fistula, abscess, pericoronitis, peri-implant inflammation, and denture-associated lesions; none were documented in any of the 17 consultations. † Composite of conservative, surgical, or periodontal treatment recommendations. ‡ 95% confidence intervals (in square brackets) are calculated using the Wilson score method and shown for these composite outcomes; for individual clinical findings and individual recommendations, only proportions are reported to preserve table readability. Note: Percentages are calculated with the respective subgroup denominator as shown in the column headings. Multiple findings or recommendations may apply to the same patient; category counts are therefore not mutually exclusive.

## Data Availability

The data supporting the findings of this study are available from the corresponding author upon reasonable request. The data are not publicly available due to institutional data protection policies governing the use of electronic medical records.
